# Critical Systematic Review of Zoonoses and Transboundary Animal Diseases’ Prioritization in Africa

**DOI:** 10.3390/pathogens10080976

**Published:** 2021-08-03

**Authors:** Serge Eugene Mpouam, Jean Pierre Kilekoung Mingoas, Mohamed Moctar Mouliom Mouiche, Jean Marc Kameni Feussom, Claude Saegerman

**Affiliations:** 1 School of Veterinary Medicine and Science, University of Ngaoundere, Ngaoundere P.O. Box 454, Cameroon; jmingoas@yahoo.fr (J.P.K.M.); mouichemoctar4@gmail.com (M.M.M.M.); 2 USAID’s Infectious Disease Detection and Surveillance Cameroon (IDDS), ICF, Yaounde P.O. Box 8211, Cameroon; 3 Cameroon Epidemiological Network for Animal Diseases (RESCAM), Directorate of Veterinary Services, Ministry of Livestock, Fisheries and Animal Industries (MINEPIA), Yaounde P.O. Box 8211, Cameroon; mfeussom@gmail.com; 4 Epidemiology-Public Health-Veterinary Association (ESPV), Yaounde P.O. Box 15670, Cameroon; 5 Research Unit in Epidemiology and Risk Analysis Applied to Veterinary Sciences (UREAR-ULiege), Fundamental and Applied Research for Animals & Health (FARAH) Centre, Faculty of Veterinary Medicine, Liege University, 4000 Liege, Belgium; claude.saegerman@uliege.be

**Keywords:** zoonoses, transboundary animal diseases, prioritization, Africa, critical systematic review

## Abstract

Background: Disease prioritization aims to enhance resource use efficiency concerning human and animal health systems’ preparedness and response to the most important problems for the optimization of beneficial outcomes. In sub-Sahara Africa (SSA), several prioritizations of zoonoses and transboundary animal diseases (TADs) have been implemented at different scales to characterize potential disease impacts. Method and principal findings: In this systematic review, we analyze the methodologies used, outcomes, and their relevance by discussing criteria required to align decision-makers’ perceptions of impacts to those of other stakeholders for different prioritization in SSA. In general, the sectorial representativeness of stakeholders for processes implemented with the support of international partners showed slight differences with the absence of local stakeholders. Whatever the tool prioritized, zoonoses were similar in general because of the structured nature of those tools in assessing decision-makers’ preferences through value trade-offs between criteria while ensuring transparency and reproducibility. However, by involving field practitioners and farmers, there were different outcomes with processes concerning only decision makers and experts who were more sensitive to infectious TADs, while the former raised parasitic disease constraints. In this context, multicriteria decision analysis-based zoonoses and TADs prioritizations involving a balanced participation of stakeholders might contribute to bridging these divergences, whatever the scale. Conclusion and significance: Prioritization processes were important steps toward building and harmonizing technical laboratory and surveillance networks to coordinate projects to address priority zoonoses and TADs at the country and/or sub-regional level. Those processes should be enhanced.

## 1. Introduction

The use of prioritization exercises for the ranking of diseases has spread from America (Canada) in 1987 [1] to other countries and continents [2]. Prioritization is the hierarchical organization of the list of pathologies by evaluating their socioeconomic and zoonotic impacts [3]. The process aims to provide decision makers with a tool to help them select the infectious risks and threats that deserve governments’ prioritization and for which appropriate preventive monitoring or control measures are needed [3]. This allows a more efficient allocation of resources (human and financial) for preparation, detection, and response measures with respect to disease prevention and control [2]. Once the goals are set, it is imperative to verify the adequacy of the current surveillance system and update it if necessary [4]. Indeed, in a context where these resources are limited, prioritization can help to concentrate resources in the right place according to one or more global objectives. The prioritization of animal diseases is applicable at the farm level with the participation of livestock farmers and animal health care professionals, in particular during health assessment. This includes identifying the dominant pathologies on farm, defining and adjusting priority mitigation measures as well as preventive measures to be implemented [5]. In addition to activities carried out at the local level, the research, prevention, and implementation of measures to combat animal diseases are mainly coordinated at national and regional levels [6]. Indeed, industrialization and economic development have encouraged the flow of so-called transboundary diseases across borders. Thus, the spread of infectious animal diseases is driven by many factors, such as the movement of humans and animals across borders, contact between wild and domestic animals, and between humans and animals [5]. Likewise, the effect on production and trade can affect not only the local economy but also the economy at the level of a group of countries and even globally. The prioritization of animal diseases and zoonoses is a complex process that must respond to health, economic, and societal priorities that are often difficult to prioritize but also must be based on a consensus between the different interested parties (breeders, veterinarians, citizens, political authorities) [7]. The prioritization of animal diseases appeared to be an essential step in optimizing the planning and allocation of limited resources that is rational, explicit and transparent. 

International bodies such as World Health Organization (WHO), World Organization for Animal health (OIE), and Food and Agricultural Organization (FAO) attach importance to the prioritization of diseases. They implemented international disease prevention and control strategies and proposed disease prioritization as a way to improve various diseases prevention and control efforts. In this regard, in 2006, the WHO published guidelines on communicable disease surveillance priorities that emphasize a prioritization role in improving disease surveillance systems at national and regional levels [8]; then, they developed a methodology to prioritize emerging infectious diseases in need of research and development [9]. Likewise, in 2014, the OIE published criteria and factors for the rational prioritization of animal diseases with public sanitary concerns for policy. Later on, many countries have initiated a prioritization process and are integrating this process as an aid in the management of animal and public health problems [7,8]. The methods used for prioritization range from qualitative to quantitative through semi-quantitative methods depending on the criteria measurement used and the type of data required [10]. Each method has its own strengths and weaknesses. Therefore, their choice and use in a specific context are subject to prior relevance and feasibility analyses in relation to prioritization purposes [2]. 

The recent growing interest and advocacy by non-profit organizations, governments, industries, and academia toward the “One Health Approach” in mitigating overlapping health problems between human, animal, and environment sectors have triggered the prioritization of zoonotic diseases in several low–middle income countries worldwide in order to coordinate preparedness and response across sectors more effectively [11]. A recent study has analyzed themes from one health zoonotic diseases workshops using the One Health Zoonotic Disease Prioritization (OHZDP) tool developed by CDC [10] in seven countries worldwide from 2014 to 2016, during the pilot phase of the tool development and testing [11]. Yet during the same period and later on, several such exercises have been carried out in other countries using the same tool or other tools such as the OIE Phylum tool [3]. Since the categorization and prioritization of diseases is not a rigid and permanent assessment requiring progressive and constant updating and iterations with respect to changes in (e.g., because it is non-exhaustive) scientific knowledge, local situation, and economic context [3], we analyze themes from animal and zoonotic diseases prioritization processes in African countries in order to draw country and regional-specific implications for the operationalization of outcomes of these processes as far as animal and zoonotic disease surveillance and control is concerned. Thus, therefore, it appears to be an imperative in the implementation and operationalization of zoonoses and animal health strategies. The aim of this review paper is to perform a critical systematic review of prioritization processes of zoonoses and transboundary animal diseases in Africa.

## 2. Results

### 2.1. Overview of Zoonosis and Transboundary Animal Disease Prioritization in Africa

The database search yielded nine relevant animal prioritization reports and 16 published papers relevant to our study. All studies were carried out and published between 2015 and 2021 (Table 1). Three animal and zoonotic disease prioritization tools were used: the OHZDP tool of the CDC [10], the OIE Phylum tool [3], and participatory epidemiology (including surveys and literature review), which is a method that promotes the active involvement and cooperation of researchers and researched populations such as livestock farmers. A total of 16 SSA countries were concerned by the present study:

- Four countries (Ethiopia, Kenya, Tanzania, and Uganda) implemented both animal and zoonotic disease prioritization processes at the national level using the Phylum and the OHZDP tool;
- Six countries (Burkina Faso, Cameroon, Cote d’Ivoire, Democratic Republic of Congo, Mali, Mozambique) were concerned only by zoonotic disease prioritization at the national level using the OHZDP tool;
- Six countries (Burundi, Djibouti, Eritrea, Rwanda, South-Sudan, Sudan) used the Phylum tool to prioritize TADs and zoonoses at the national level.

A local prioritization of animal diseases (participatory epidemiology) concerning one of the sixteen countries above (Ethiopia) and regional prioritizations of zoonoses and animal diseases in West and East Africa were retrieved as well.

For regional and national prioritization, workshops were specifically conducted, and all processes aimed at identifying and prioritizing either only zoonotic diseases or both zoonoses and animal diseases. Subject expert matters (or trained facilitators) contributed to the processes whatever the tool used following standard procedures previously described. 

### 2.2. Prioritization Processes by Stakeholders

**Figure 1:**
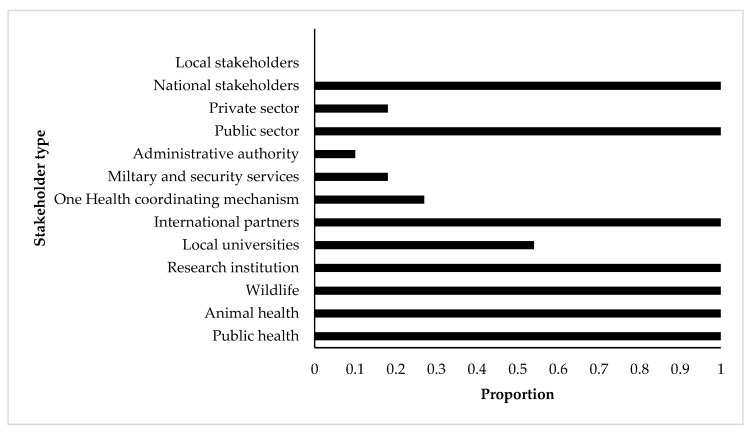
Participation proportion of stakeholders involved in the prioritization processes of zoonotic diseases by the CDC One Health Zoonotic Disease Prioritization tool for 10 SSA countries (Burkina Faso, Cameroon, Cote d’Ivoire, Democratic Republic of Congo, Ethiopia, Kenya, Mali, Mozambique, Tanzania, and Uganda). *X*-axis shows the proportion of participation of each stakeholder type (*Y*-axis) in the included prioritization processes.

During the OHZDP process at the national level, participating members were grouped by their agencies and voted on the ranking or weight applied to each criterion before conducting a final ranking of diseases. Participants belonged to different national sectorial or administrations including public health (all countries), animal health (all countries), wildlife and environmental health (all countries), research institutions (all countries), local universities (half the countries), international partners, one health coordinating institutions (one-third of the countries), and military and security services (one-fifth of the countries). The private sector was involved only in two out of the ten concerned countries, and the participants were from central or national administration for all countries (Figure 1). The participating stakeholders for the regional prioritization came from the 15 countries and their institutions were similar to the one from the national OHZDP processes. 

**Figure 2:**
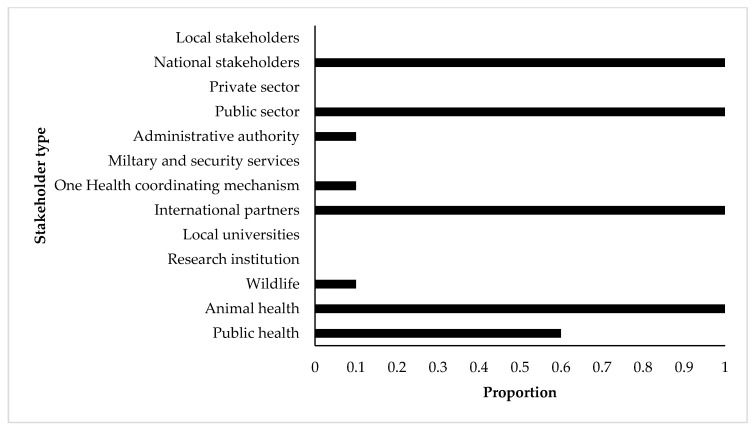
Participation proportion of stakeholders involved in the prioritization processes of zoonotic diseases by the OIE Phylum tool for 10 SSA countries (Burundi, Djibouti, Eritrea, Ethiopia, Kenya, Rwanda, South-Sudan, Sudan, Tanzania, Uganda). *X*-axis shows the proportion of participation of each stakeholder type (*Y*-axis) in the included prioritization processes.

The prioritization of TADs and zoonoses using the Phylum tool was carried out by senior experts from the national veterinary departments (all countries), public health departments (three-fifths of the countries), and international partners (all countries). Wildlife, administrative authorities, and One Health coordinating institutions were each involved in only one of the ten countries. In all countries, participants were mainly from the public sector and from central or national administration for all countries (Figure 2).

### 2.3. Zoonoses and Transboundary Animal Diseases Prioritization Criteria Chosen by Countries

For the OHZDP process, irrespective to the assigned weight, the most frequent disease ranking criteria used were (Table 2):

- Severity of disease in humans (all countries),
- Availability of interventions (i.e., vaccines and/or medical treatment) (all countries),
- Economic, environmental, and/or social impact (9/10 countries),
- Presence of disease in country and/or region (8/10 countries),
- Epidemic/pandemic potential (and/or sustained transmission in humans) (7/10 countries).

With the Phylum tool, the importance of the listed diseases was assessed based on the scoring of selected specific criteria including local economic impact, local public health impact, local societal impact, local environmental impact, and the local feasibility, economic, societal, and environmental impact of control measures. The assessments considered both present and absent diseases (Phylum). For each listed disease, the tool uses expert opinion bases on data from public health and veterinary services, statistics from health information systems (e.g., World Animal Health Information System (WAHIS) from OIE, FAO (Empres-I), AU/IBAR (Aris) OMS) for the prioritization of TADs and zoonosis. The analysis of a disease is made in two sequential steps: first, a global characterization of the disease, aiming at assessing the different above-mentioned impacts within the region, independent of any particular local context [3]. Depending on the selected criteria, the characteristics of the studied diseases, and local contingencies, the scores may not be homogenous between the different modules applied, therefore requiring standardization of the results. Then, results are subjected to discussions (subject matter expert opinion) by stakeholders in the country who provide their inputs and a final list of prioritization [3]. Five countries (Burundi, Djibouti, Eritrea, South Sudan, and Sudan) provided detailed lists of justifications used for scoring diseases in each of the seven criteria used for classification by the Phylum tool (Table 3). Based on the local economic impact, the main justifications used were trade and export bans (all the five countries), high morbidity, high mortality, and hindering industry (four countries out of the five for each justification). For public health impact, the zoonotic status of the disease (all five countries), high cost of control and prevention for public health (four out of five countries), export and trade bans (three out of five countries) were the main justifications. Considering the social impact of diseases, the zoonotic status of the diseases (all the five countries), mortality in animals, mortality/case fatality in humans, negative impact on pastoralists such as poverty (four out of five countries each) were mainly used for disease scoring. The local environmental impact justifications used for disease scoring were mainly biological and chemical contamination of the environment (all five countries), and the disposal of infected and dead animals (four out of five countries). For the feasibility of control measures, the extend of public importance and implications, mortality rate, vaccination constraints (three out of five countries each) were considered for disease scoring. Trade bans (four out of five countries), the endemic status of the disease, highly contagious diseases, the high cost of control and prevention for public health, human economic impacts, zoonotic status of diseases (three out of five countries each) were used as justification for the scoring of diseases based on the local economic impact of control measures. Control measures’ constraints (all five countries) and wildlife susceptibility (three out of five countries) were the main justifications considered for scoring the diseases according to the local societal and environmental impact of control measure criterion.

### 2.4. Zoonoses and Transboundary Animal Diseases Ranking

As a result of the OHZDP tool’s ranking process, the ten countries listed on average 38.5 zoonotic diseases (range: 11–48) of which 5.7 (range: 5–7) and 0.6 (range 0–1) endemic and exotic zoonoses were prioritized, respectively (Table 4). Eighteen zoonoses or syndromes were ranked as priority zoonotic diseases (Table 5). Of those, rabies (*n* = 10), anthrax (*n* = 7), brucellosis (*n* = 7), zoonotic influenza virus (*n* = 7), and hemorrhagic fever (Ebola/Marburg) (*n* = 6) were the top five most prioritized zoonoses amongst the studied countries. Of that ranking zoonotic influenza virus (*n* = 2), hemorrhagic fever (Ebola/Marburg) (*n* = 2), trypanosomiasis (*n* = 1), and dengue (*n* = 1) were the ones ranked as exotic zoonotic diseases. Within the ECOWAS region, 30 zoonotic diseases were listed, and seven (anthrax, rabies, viral hemorrhagic fevers (Rift valley fever, Ebola, Crimean Congo hemorrhagic fever, Marburg), zoonotic influenzas, zoonotic tuberculosis, trypanosomiasis, and yellow fever) were selected as priority zoonoses. Eleven, five, and two zoonoses prioritized by the ten countries were caused by viruses, bacteria, and parasites, respectively. 

Amongst the ten countries that implemented the Phylum prioritization process, 15.90 (range: 11–23) of which 8.80 (range: 6–12) and 3.50 (range 2–6) were endemic and exotic TADs and zoonoses were prioritized respectively in each country (Table 4). Nine endemic TADs and nine zoonosis or syndromes were prioritized (Table 5). Of those, the top five most ranked endemic TADs were foot and mouth disease (*n* = 10), peste des petits ruminants (*n* = 9), contagious bovine pleuropneumonia (*n* = 8), New Castle disease (*n* = 7), and contagious caprine pleuropneumonia (*n* = 5). Brucellosis (*n* = 8), rabies (*n* = 7), and tuberculosis (Mycobacterium bovis) (*n* = 6) were zoonoses ranked by the most countries. In addition, exotic priority diseases were ten TADs and four zoonoses where specific serotypes of foot and mouth disease (*n* = 9) was the main exotic TADs ranked (Table 5). Highly pathogenic avian influenza (*n* = 10) and Rift valley fever (*n* = 5) were the most prioritized zoonoses when being exotic to the country. Endemic TADs prioritized were caused by viruses (*n* = 6), bacteria (*n* = 2), and parasites (*n* = 1). Three, four, and two endemic zoonosis were caused by viruses, bacteria, and parasites, respectively. Eight and two exotic TADs prioritized were caused by viruses and bacteria, respectively. All exotic zoonoses (*n* = 4) prioritized were caused by viruses.

## 3. Discussion

The need of achieving the optimal benefit in tackling animal and human health challenges or constraints has prompted the prioritization of diseases as a strategic process ensuring efficient use of limited resources to target the most important problems. The process of prioritizing animal diseases and zoonoses has been implemented previously in different locations, with different magnitudes and purposes in South and North America [37,38], Europe [39,40,41,42], Asia, and Africa [2] (Table 1). Those studies have ranked various types of diseases such as zoonotic and foodborne diseases, based on several measured and weighed criteria that describe or characterize potential disease impacts [2]. This study has reviewed recent prioritization processes carried out in SSA with various tools used at different scales in the context of “One Health”. A previous study has included five SSA countries in such an analytical review of prioritization processes together with two other countries from South Asia [11] using the OHZDP tool. However, several animal diseases prioritization processes have been implemented during the same timeframe or later on using the same tool or other diseases prioritization tools. In this study, the prioritization of TADs and zoonoses has been considered at the country level (*n* = 16) using previously developed prioritization tools. In addition, regional (West and East Africa countries) and local (District) level prioritization processes were also taken into consideration. To the best of our knowledge, this study is one of the few of its kind to gather knowledge generated through several prioritization processes using different tools in SSA whereas previous synthetic works focused on analyzing data generated through the processes using the same tool [11]. 

For the prioritization of animal diseases and zoonoses, it has previously been argued that using an interdisciplinary team of participants (facilitating and voting) who stay neutral, unbiased, and do not focus on their specific sector, affiliation, or area of expertise enables voting members’ voices to be heard and recognized [2,11]. In this study, we found that the OHZDP tool thrives to meet the interdisciplinary and multisectorial compliance throughout the process more than the Phylum tool does. The other processes gave fewer details as far as experts involved in the process of prioritization were concerned. Yet it must be pointed out that the animal and human health private sectors and local or field professionals were less involved during both processes, making them elitist and top–down decision-making approaches whereby governmental sectors and top ranked professionals at the central or national level identify and classify priority diseases and impose them on private practitioners and field or peripheral professionals. This can implicitly have negative implications at the operationalization phase of the use of the priority disease lists and the implementation of mitigating actions (poor community engagement). To cope with the challenge of dealing with several pathogens posing specific threats to animal and public health, all prioritization processes required an extensive literature review. The latter allowed the identification of diseases relevant to classification and sound evidence-based prevention and control measures as well as efficient resource allocation [2,39]. Indeed, in this study, there was a wide variation of disease list lengths (number of listed diseases) between countries whatever the prioritization method used. Prioritization processes were held during a workshop (or focus group discussion and surveys) following a preparation period lasting several months and deeply relying on local partners’ engagement and implication of technical and financial partners [3,10,35,36]. Criteria identification and/or weighing or disease classification based on previously identified criteria were performed by the selected participants. Despite the differences existing between the different prioritization processes, economic, human health, societal, and environmental impact are the most used categories (with specific weighing and scoring justifications), as diseases are responsible for a variety of overlapping impacts than span over these different categories [43]. In fact, there are several drivers of disease impacts, including for instance climatic, biophysical, anthropogenic, and epidemiological factors resulting in a wide variation of disease importance according to species, scale (local, national, regional, global), and the perception of stakeholders [44]. Thus, defining disease importance is not a straightforward and trivial process as shown by past and recent development of disease prioritization processes [2,42]. For most tools, no matter the disease types of interest (zoonoses or animal diseases), the selected criteria mainly targeted diseases known to be present in the country except for the Phylum tool, where distinction is made between endemic and exotic diseases during the prioritization process [3].

Whatever the tool used at the country level (CDC OHZDP or OIE Phylum), zoonotic diseases that were most frequently prioritized were rabies, brucellosis, hemorrhagic fevers (Marburg/Ebola), anthrax, Rift Valley fever, and zoonotic tuberculosis. For public health, this consensus on prioritized zoonoses also shows how these endemic diseases are important at the country and continental level. Rabies is an acute and progressive viral encephalitis causing approximately 59,000 human deaths annually in Africa and Asia [45,46], and it is responsible for more than 1.74 disability-adjusted life years (DALYs) lost each year, which makes it an important yet neglected disease in Africa and Asia [47]. Brucellosis is a group of zoonoses caused by bacteria of the genus Brucella, which is a widespread problem in Africa [48,49,50] having negative public health and causing important animal production and economic losses [51]. Hemorrhagic fevers (Ebola viral disease and Marburg disease) constitute major public health issues in SSA where the 2870 cases (2232 Ebola cases) documented between June 1967 and June 2011 with 1503 deaths (for Ebola) were by far lower than the 22,859 cases and a total of 9162 deaths reported in a very short time during the 2014–2015 outbreaks [52,53], resulting in economic growth losses ranging from 4.9 to 18.7% in affected countries [54]. Anthrax is a dreadful disease occurring in most SSA countries [55] due to a Gram-positive, rod-shaped, and spore-forming bacterium affecting primarily wild and domestic herbivores, resulting in high mortality rates accompanied with an important human health [56] and bioterrorism risk when inhalation of the etiological agent occurs in humans, causing severe respiratory symptoms associated with high mortality rates similar to the 2001 anthrax attacks in the United States [57]. Rift Valley fever is a vector-borne viral disease that affects mainly domestic ruminants and occasionally humans in SSA [58,59] which is listed as one of the priority diseases in the WHO Blueprint list because of its epidemic potential and lack of effective countermeasures. Due to the extensive livestock production systems operating in most African countries millions of people are still at risk of contracting zoonotic tuberculosis from a range of mycobacterium species infecting animals [60,61,62,63]. Foot and mouth disease (FMD), peste des petits ruminants (PPR), contagious bovine pleuropneumonia (CBPP), Newcastle diseases, and contagious caprine pleuropneumonia were the most prioritized endemic TADs causing huge production and economic losses, although diagnostic, surveillance, and control measures exist in most of the countries considered [64,65,66,67,68]. Estimates of the annual economic impact of FMD in terms of direct production losses and vaccination in endemic regions amount to USD 6.5–21 billion [66], in addition to indirect social costs [69]. Moreover, in Niger, economic impacts of FMD were estimated at outbreak (herd) level at 499 euros [70]. For PPR, based on a 15-year vaccination program with total discounted costs of USD 2.26 billion, a net benefit of USD 74.2 billion is projected [67]. Annually in 12 SSA countries, the cost of losses due to morbidity and mortality resulting from CBPP-affected animals amounted to USD 37.8 million, while the total economic cost was estimated at USD 56.5 million or an average of USD 4.41 million per country [64]. Contagious caprine pleuropneumonia (CCPP) is an important respiratory disease of small ruminants that causes huge losses in Africa and Asia with a global burden estimated at USD 507 million annually [68]. Outbreaks of virulent Newcastle disease (ND) in poultry is often associated with high mortality (up to 100%), being therefore a major constraint on the productivity of village chicken flocks in Africa [65,71,72]. 

In total, four parasitic diseases (three parasitic zoonoses and one animal disease) were ranked between the 18 top diseases, suggesting that decision makers and experts’ perception of the impact of bacterial and viral infections is higher than the one of parasitic diseases. However, developed countries that have succeeded in controlling most of the diseases prioritized in Africa are mainly focused on the introduction of emerging and re-emerging infectious and food-borne (bacterial and viral) diseases [39,40,41,42,73]. Risk perception of emerging and re-emerging infectious diseases varies according to stakeholders [74]. Livestock keepers in Africa are rural poor pastoralists, and with regard to priority diseases, it has been found that the perception of the poor themselves varied widely from expert opinion and para-veterinarians and community animal health workers that share the day-to-day life of farmers [75]. Thus, at the local level despite not being diseases properly speaking, ectoparasite infestations were top ranked, followed by known diseases or syndromes such as contagious bovine pleuropneumonia (CBPP), foot and mouth disease (FMD), blackleg, bloody diarrhea, and pasteurellosis [35]. Similarly, at national/regional levels, the top constraints that emerged from a prioritization process including three approaches (literature review, expert workshops, and para-veterinarian practitioner surveys) were endo/ectoparasites, FMD, brucellosis, peste des petits ruminants, Newcastle disease, avian influenza, contagious caprine pleuropneumonia, contagious bovine pleuropneumonia, mastitis, reproductive disorders, and nutrition constraints [36]. The listing of endo/ectoparasite infestations and nutrition constraints as major animal health problems in farming communities shows that more research is required to better understand and bridge the differences between decision makers and the farmers about their perception of disease impacts [75] for efficient animal and zoonotic disease prevention and control at all levels. In this regard, community animal health workers and local public health professionals could play a great role. Thus, as far as stakeholders are concerned, in addition to area of expertise and multisectoral considerations, prioritization processes should go down to the field or community health practitioners and representative of farmers. Such an approach should make sure to minimize stakeholder-driven prioritization biases, and funding/research priorities should align with improving the welfare of smallholder livestock keepers while taking into consideration human and environmental health interests [36,76] by therefore mitigating subjectivity (individual or group), enhancing transparency needed for disease prioritization exercises, and combining both natural and social sciences research during prioritization processes [5]. Yet disease prioritization processes should be updated as new information affecting drivers or criteria measurements arises [2]. Only then through cooperation and coordination can the effectiveness of disease research and control programs (pooling of resources, knowledge and means of control, comprehensive understanding of the impact of diseases, etc.) can be improved.

At the time of this study, five countries (Ghana, Rwanda, Senegal, Sierra Leone, and South Africa) that prioritized zoonotic diseases using the OHZDP tool had not published their reports yet, and all Phylum country prioritization processes were considered. Thus, the prioritization processes reviewed in this study and carried out at the regional and national level are in no way exhaustive though representative and may be only partly useful at the local level where a full list of disease constraints should be identified based on production systems. In this regard, prioritization processes should be implemented in a standardized format that can be flexibly used whatever the scale and stakeholders involved such as multicriteria decision analysis (MCDA), which is a set of methods from decision science that have been recently used for disease prioritization [41,77]. However, these prioritization processes were important steps toward building and harmonizing technical laboratory and surveillance networks to coordinate projects addressing priority zoonoses and TADs at the country and/or sub-regional level while improving sub-regional and regional legislative frameworks for prevention and control initiatives at the human, animal, and environment interface. Those processes are already being supported by international organization such as the FAO, OIE, and WHO [78] and should be enhanced. 

## 4. Materials and Methods

**Figure 3:**
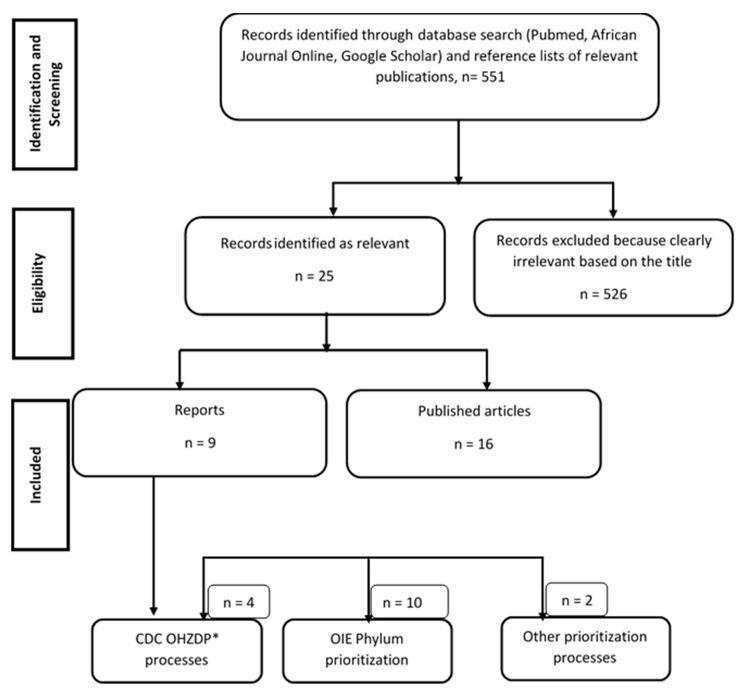
Systematic search of papers and reports on zoonosis and transboundary animal disease (TADs) prioritization processes in Africa before February 2021. * OHZDP: One Health Zoonotic Disease Prioritization.

We attempted to gather details of all published (journal articles, reports published in English and French languages) studies and reports involving animal and zoonotic disease prioritization in sub-Sahara Africa (SSA) (Figure 3). Country-specific reports of animal and zoonotic disease prioritization carried out using the One Health Zoonotic Disease Prioritization Process (OHZDP) designed by the CDC [10], the Phylum tool of OIE [3], surveys, and participatory epidemiology were also retrieved online for data extraction. African countries that had not published their prioritization reports before February 2021 were not included in the present study. We excluded all publications/reports not concerning animal and zoonotic diseases prioritization in SSA. We searched through 3 databases: PubMed, Google Scholar, and AJOL (African Journals OnLine). As search terms, we used animal and/or zoonotic and disease and prioritization in Africa, because our interest is animal disease prioritization no matter the time, scale, and species but limited to SSA countries. A set of data extracted from each paper/report concerned participant institutions, selected prioritization criteria, criteria weight, diseases selected for prioritization, and disease normalized scores. Participants’ profiles with respect to their institutions/sector of activity were descriptively analyzed. Criteria identified and used for prioritization were aggregated, and their mean weights were compared to analyze their influence on the classification. Data were aggregated according to the prioritization method used and analyzed using Microsoft Excel (Microsoft, Redmond, WA, USA).

## 5. Conclusions

Animal husbandry in SSA is strongly constrained by diseases that have various economic and public health impacts. In this critical review, we have analyzed several zoonosis and animal disease prioritization processes carried out in SSA. By aggregating data for tools that were used several times for prioritization, we characterized criteria and participating stakeholders. Then, diseases that have the greatest impact on livestock production and public health according to stakeholders were identified and ranked. There was a general agreement of priority zoonoses whatever the tool used for prioritization except for some diseases or syndromes seen as important for farmers or practitioners. The limitations of each prioritization process were discussed and ways of improvement were presented. Few research studies have focused on synthetizing disease prioritization outcomes from diverse prioritization methods and questioning the operationalization of the surveillance and control of the prioritized diseases. Thus, in SSA, the operationalization of outcomes from the prioritization of zoonoses and TADs at the regional, country, local, and farm levels can be certainly improved by involving all stakeholders particularly at the local level through standardized prioritization methods such as multicriteria decision analysis.

## Figures and Tables

**Table 1:** Location, scales, purposes, and tools used for animal and zoonotic disease prioritization in SSA countries.

Region–Country–Locality	Year	Scale	Purpose of Prioritization	Methods	Reference
Burkina Faso, Cameroon, Cote d’Ivoire, Democratic Republic of Congo, Ethiopia *, Kenya *, Mali, Mozambique, Tanzania *, Uganda *	2015–2018	National	To identify zoonotic diseases of greatest national concern for the concerned countries using equal input from representatives of human health, animal resources, and the environment	Semi quantitative, CDC OHZDP tool	[12,13,14,15,16,17,18,19,20,21,22,23]
Burundi, Djibouti, Eritrea, Ethiopia *, Kenya *, Rwanda, South-Sudan, Sudan, Tanzania *, Uganda *	2015–2016	National	To identify, prioritize, and categorize the key transboundary animal diseases and zoonoses for public policy and animal health programs at the national level	Semi quantitative, OIE Phylum tool	[24,25,26,27,28,29,30,31,32,33]
**ECOWAS** (Benin, Burkina Faso Cape Verde, Côte d’Ivoire, Gambia, Ghana, Guinea, Guinea Bissau, Liberia, Mali, Niger, Nigeria, Senegal, Sierra Leone, Togo	2019	Regional	To use a multisectoral, One Health approach to identify zoonotic diseases of greatest regional concern for ECOWAS (13 countries)	Semi quantitative, CDC OHZDP tool	[34]
Ethiopia * (districts of Lalibela, Sekota, and Ziquala)	2016–2018	Local	To identify and prioritize primary cattle disease with the aid of participatory epidemiology tools (i.e., focus group discussions and questionnaires)	Participatory epidemiology tool	[35]
**West Africa**: Senegal, Mali, Ghana, Burkina Faso, Cote D’Ivoire, Chad, Togo, Benin, Nigeria, Sierra Leone. **East Africa**: Tanzania *, Kenya *, Uganda *, Ethiopia *, South Sudan, Malawi, Mozambique, Zambia	2019	Regional	To prioritize animal health needs in East and West Africa and South Asia to identify diseases and syndromes that impact livestock keepers	-Systematic literature Review -Expert workshops -Veterinary practitioner survey	[36]

Legend: * Countries that implemented animal and zoonotic disease prioritization using the CDC OHZDPT (One Health Zoonotic Disease Prioritization Process) and the OIE Phylum tool; ECOWAS: Economic Community of West African States.

**Table 2:** Disease ranking criteria chosen by country during One Health Zoonotic Disease Prioritization workshops in ten SSA countries ^a^.

Disease Ranking Criteria	No. Countries	Average Assigned Weight (Range)
Economic, environmental, and/or social impact	9	0.170 (0.105–0.210)
Economic impact only	0	
Economic and/or social impact	5	
Economic, environmental, and/or social impact	4	
Availability of interventions (i.e., vaccines and/or medical treatment)	10	0.174 (0.130–0.206)
Epidemic/pandemic potential (and/or sustained transmission in humans)	7	0.201 (0.170–0.220)
Human-to-human transmission potential	7	
History of previous outbreaks	0	
Severity of disease in humans	10	0.231 (0.180–0.350)
Case-fatality rate	8	
Morbidity and/or mortality rate	2	
Presence of disease in country and/or region	8	0.232 (0.170–0.330)
Human and/or animal cases of illness reported in country and/or region	6	
Human or animal disease prevalence and distribution in country	2	
Laboratory capacity/diagnostic testing capacity	2	0.151 (0.143–0.160)
Existing multisectoral collaboration	1	0.170 (NA)
Bioterrorism potential	3	0.102 (0.040–0.187)
Mode of transmission	1	0.200 (NA)

Legend: ^a^ Countries: Burkina Faso, Cameroon, Cote d’ Ivoire, Democratic Republic of Congo, Ethiopia, Kenya, Mali, Mozambique, Tanzania, Uganda; NA: not available.

**Table 3:** Justifications used in the literature for the Phylum prioritization of transboundary animal diseases and zoonoses in five SSA’s countries ^a^.

Classification Criteria							
Economic Impact		Human Health Impact		Societal Impact		Environmental Impact	
Justification	%	Justification	%	Justification	%	Justification	%
Export and trade bans	100	Zoonotic disease	100	Zoonotic disease	100	Contamination of the environment	100
High mortality	80	High cost of control and prevention for public health	80	Mortality in animals	80	Disposal of animals	80
High morbidity	80	Export and trade bans	60	Mortality/case fatality in humans	80	Cleaning and disinfecting costs	60
Hindering industry	80	Negative impact on tourism	40	Negative impact on pastoralists (poverty)	80	Risk for wildlife	60
High cost of animal disease control and prevention	60	Number of reported cases	40	Impact on consumption habit	60		
High cost of control and prevention for public health	60	Mode of transmission	40	Morbidity in animals	60		
Cost if introduced	40	Mortality/case fatality	40	Production lost	60		
Highly contagious	40	Bioterrorism potential	20	Social stress	60		
Production lost	40	Effect on livelihood	20				
Zoonotic disease	40	Endemic zoonoses	20	High cost of animal disease control and prevention	40		
Endemic disease	20	Food borne	20	High cost of control and prevention for public health	40		
Food borne	20	Highly contagious	20	Number of reported cases	40		
Negative impact on pastoralists (poverty)	20	Movement restriction	20	Bioterorrism potential	40		
Negative impact on tourism	20			Export and trade bans	20		
Great public importance and implications	60	Trade bans	80	Control measures’ constraints			100
High mortality	60	Endemic	60	Wildlife susceptibility			60
Vaccine constraints	60	Highly contagious	60	Zoonotic disease			40
Culling constraint	20	High cost of control and prevention for public health	60	Morbidity			40
Food security issues (poultry meat and egg, beef, milk)	20	Human economic impacts	60	Mortality			40
Testing constraints	20	Zoonotic disease	60	Consumption habit			20
Local and international trade	20	Tourism	40	Increased production costs			20
Movement restriction feasibility	20	Increased production costs	20	Environmental contamination			20
Vector control feasibility	20	Control constraints					
Zoonotic disease	20						

Legend: ^a^ Burundi, Djibouti, Eritrea, South Sudan, Sudan.

**Table 4:** Number of animal diseases and zoonoses listed for prioritization in SSA countries.

Country	OHZDP * Tool				Phylum Tool			
	No Listed Diseases	No Prioritized Endemic Diseases [A]	No of Prioritized Exotic Diseases [B]	Total [A] + [B]	No Listed Diseases	No Prioritized Endemic Diseases [C]	No Prioritized Exotic Diseases [D]	Total [C] + [D]
Burkina Faso ^a^	41	4	1	5				
Burundi ^b^					23	10	6	16
Cameroon ^a^	41	4	1	5				
Cote d’Ivoire ^a^	40	5	1	6				
Democratic Republic of Congo ^a^	11	6	0	6				
Djibouti ^b^					15	6	0	6
Eritrea ^b^					16	7	4	11
Ethiopia ^c^	43	5	0	5	16	7	3	10
Kenya ^c^	36	5	0	5	15	13	2	15
Mali ^a^	38	5	0	5				
Mozambique ^a^	48	6	1	7				
Rwanda ^b^					16	10	6	16
South-Sudan ^b^					15	12	3	15
Sudan ^b^					11	6	6	12
Tanzania ^c^	39	5	1	6	16	7	2	9
Uganda ^c^	48	6	1	7	16	10	3	13
Average (S.E. **)	38.5 (3.30)	5.1 (0.23)	0.6 (0.16)	5.7 (0.26)	15.9 (0.92)	8.8 (0.80)	3.5 (0.64)	12.3 (1.05)

Legend: * OHZDP: One Health Zoonotic Disease Prioritization; S.E. **: standard error of the mean; ^a^: countries that prioritized zoonotic diseases using the OHZDP tool; ^b^: countries that prioritized transboundary animal diseases and zoonoses using the OIE Phylum tool; ^c^: countries that carried out both prioritization processes; No: Number.

**Table 5:** Top zoonoses and transboundary animal diseases (TAD) prioritized by the OHZDP and Phylum Tools in SSA.

Type	Disease or Health Condition	Causative Agent			No of Countries	
			OHZDP Tool ^a^		Phylum Tool ^b^	
			Endemic	Exotic	Endemic	Exotic
Zoonosis	Rabies	Virus	10		7	1
	Anthrax	Bacteria	7		1	
	Brucellosis	Bacteria	7		8	
	Zoonotic influenza	Virus	5	2	1	9
	Hemorrhagic fever (Ebola/Marbug)	Virus	4	2		2
	Rift Valley fever	Virus	4		3	5
	Trypanosomiasis	Parasite	3	1	1	
	Zoonotic tuberculosis (*M. bovis*)	Bacteria	3		6	1
	Lassa	Virus	2			
	Salmonellosis	Bacteria	2			
	Arboviral diseases *	Virus	1			
	Crimean-Congo hemorrhagic fever	Virus	1			
	Dengue	Virus		1		
	Echinococcosis	Parasite	1			
	Leptospirosis	Bacteria	1			
	Monkey pox	Virus	1			
	Plague	Virus	1			
	MERS-CoV **	Virus	1			
	Swine erysipelas				1	
TAD	Foot and mouth disease	Virus			10	6
	Peste des petits ruminants	Virus			9	1
	Contagious bovine pleuropneumonia	Bacteria			8	2
	New Castle disease	Virus			7	
	Contagious caprine pleuropneumonia	Bacteria			5	
	African swine fever	Virus			4	1
	Lumpy skin disease	Virus			4	
	Sheep and goat pox	Virus			3	1
	East coast fever	Parasite			2	
	Porcine cysticercosis	Parasite			1	
	Classical swine fever	Virus				1
	PRRS ***	Virus				1
	Camel pox	Virus				1

Legend: * e.g.: yellow fever and West Nile disease; ** MERS-CoV: Middle East respiratory syndrome; *** PRRS: porcine reproductive and respiratory syndrome; ^a^: countries that used the OHZDP tool (Burkina Faso, Cameroon, Cote D’ Ivoire, Democratic Republic of Congo, Ethiopia, Kenya, Mali, Mozambique, Tanzania, Uganda); ^b^: countries that used the Phylum tool (Burundi, Djibouti, Eritrea, Ethiopia, Kenya, Rwanda, South-Sudan, Sudan, Tanzania, Uganda); No: number.

## Data Availability

Secondary data generated from reports and published papers and supporting the findings of this study are available from SEM upon request.
